# Geographical variations of early age sexual initiation among reproductive-age women in Ethiopia: evidence from EDHS 2016

**DOI:** 10.1186/s13690-020-00411-4

**Published:** 2020-06-01

**Authors:** Araya Mesfin Nigatu, Abraham Yeneneh Birhanu, Berhanu Fikadie Endehabtu

**Affiliations:** Department of Health Informatics, Institute of Public Health, University of Gondar, P.O.Box 196, Gondar, Ethiopia

**Keywords:** Early sexual initiation, Geographical variations, Ethiopia

## Abstract

**Background:**

The early age of sexual initiation contribute a lot for various risks such as mistimed pregnancy followed by insecure termination, developing fistula and contracting sexually transmitted infections which are currently the major public health concerns for low-income countries. Therefore, the purpose of this study was to detect spatial clusters and identify factors associated with an early age sexual initiation of women in the reproductive age group.

**Methods:**

We used a population-based nationwide representative Ethiopian Demographic and Health Survey (EDHS) 2016 data.. A total of 12,033 respondents of reproductive age (15–49 years) women who had at least one event of sexual intercourse was retrieved and included for the analysis. Spatial cluster detection and autocorrelation analysis were also done to explore the patterns of early age sexual initiation.

**Results:**

The median age at first sexual intercourse among respondents was 16 (±3.3) years and more than half (66.2%) had their first sexual intercourse before the age of 18 years. The spatial variations of the age of sexual initiation was nonrandom and clustered with a Moran’s I = 0.413 (*P*-value < 0.001). In addition, five significant spatial clusters were also identified. Moreover, the probability of starting sex at an earlier age was associated with the respondent’s residence, marital status, educational attainment and wealth index.

**Conclusion:**

This study found a higher proportion of an early age sexual initiation of women. Respondent’s residence, marital status, educational attainment and wealth index were significantly associated with early sexual initiation. The SaTScan analysis identified five statistical significant spatial clusters which indicate that there were geographical variations. Therefore, integrated interventions focusing on the identified high spot clustered areas are recommended to reduce early age sexual initiation.

## Background

According to World Health Organization (WHO) report, annual number of maternal deaths and life time risk had showed significant improvement. Maternal death declined by 43% and lifetime risk improved from one in 73 to one in 180 [1]. However, developing regions still account nearly 99% of the global maternal deaths in which sub-Saharan Africa alone accounts for roughly 66%, followed by Southern Asia 22% [1]. From 20 million unsafe abortions done worldwide, 68,000 of maternal deaths were reported; from which teenage girls account for 14% [2].

Globally, 1.2 billion worldwide population are adolescents aged from 10 to 19 years; more than 1.1 million adolescent groups died of preventable causes related to pregnancy and childbirth complications which is the leading cause of death for 15–19 year’s old age groups of girls [3]. Among adolescents with age less than 17 years; 30% of them have had sex and 252,000 pregnancies occur annually [4].

In Ethiopia, there are nearly 6 million women who are within the age group of 15–19 accounting for 12% of the total female population with extremely limited media access (26% report at least weekly exposure to radio, 18% of television and 9% to newspapers); consequently, 39% had first sex before age 18 and 28% of recent births to women younger than 20 were unplanned [5].

According to EDHS 2011 report, 62% of women had sexual intercourse before the age of 18 years [6].

A number of findings pointed out that, early age of sexual initiation is currently a progressive important concern for sexually active young adolescents. Youngsters who started early age sexual intercourse developed more risk behaviors which would have the likelihood of having multiple sexual partner that could lead them to many negative health outcomes (such as: unwanted pregnancies, unsafe abortion, vaginal fistula, sexually transmitted infections) and substantial socio-economic burdens [6–9]. There are many factors (intrinsic and extrinsic) which enables women to be engaged in early sexual initiation such as; low awareness on how and when to use contraceptive methods, self-desire to experience sex, social fitness, economic challenges, chewing Khat, drinking alcohol, watching pornographic materials, being less in parents, media content and social/religious ceremonies have been playing a major role [6–9]. Many studies have been conducted by concentrating on the demographic, socioeconomic, obstetric factors and reasons of adolescents to be engaged in early sexual intercourse [8–10], whereas little is known, about the geographical variations related to the issue. Therefore, the purpose of this study was to examine geographical disparities, and to identify significant predictors of an early age sexual initiation among reproductive age women in Ethiopia.

## Methods

### Study design and setting

A cross-sectional study was conducted to detect geographical clusters and determine factors associated with early age sexual initiation among reproductive-age women from EDHS 2016 data. The study was conducted in Ethiopia (3^o^-14^o^N and 33^o^ – 48°E), located at the horn of Africa. Governmentally, the country is divided into nine regional states and two city administrations [11]. Each region is sub-divided into zones, districts, towns, and kebeles (the smallest administrative units).

### Data

The data for this study were retrieved from the DHS program authorized database. The survey is usually conducted at five-year intervals in a country. The country has undertaken four consecutive DHS surveys in (2000, 2005, 2011 and 2016). The Ethiopian DHS was planned to have estimates from the nine regional states and two city administrations. Geographical location data (latitude and longitude coordinates) were also taken from selected enumeration areas. The survey data sets and location data were salvaged through the web page of the international demographic health survey program after subscription and being an approved user.

### Sampling technique and sampling size

**Fig. 1:**
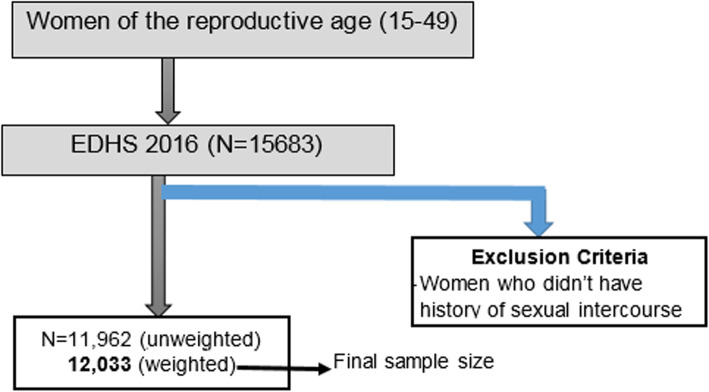
The exclusion procedures to identify the final sample size in Ethiopia, EDHS 2016

A stratified two-stage cluster sampling technique using a national representative population-based survey was employed. The data were collected from 645 enumeration areas (EAs) (202 urban and 443 rural areas) independently in each stratum of the two stages using systematic sampling with probability proportional to size. After applying the weighting technique, a total of 12,033 women of reproductive-age (15–49 years) who had at least one occurrence of sexual intercourse was retrieved and included for the analysis. Spatial cluster detection and autocorrelation analysis were also done to discover the patterns of early age sexual initiation (Fig. 1).

### Key variables and measurements

#### Dependent variable

The study variables were grouped into dependent and independent variables. The dependent variable was early age sexual initiation, categorized dichotomously as “Yes/No” variable. Respondents who were engaged in sexual intercourse before the age of 18 were categorized as “Yes” and those who didn’t as “No.”

#### Independent variables:

- **Socio-demographic variables**: Current respondents age, residence, region, religion wealth index, women’s education, working status, marital status, age at first marriage
- **Sexual characteristics variables**: Pregnancy, abortion, number of children ever born

### Data collection procedures and period

Briefly, data were collected by visiting households and conducting face-to-face interviews to obtain information on demographic characteristics, socioeconomic status, sexual and reproductive history, starting from January 18, 2016, to June 27, 2016 [11].

### Operational definition

Early age sexual initiation was defined as the experience of first intercourse before 18 years of age [9, 11].

### Statistical analyses

#### Descriptive and inferential analysis

All variables in the DHS data for this analysis were given weight to adjust differences in the probability of selection and to adjust non-response in order to produce the proper representation using SPSS version 20. Descriptive statistics like frequencies, percentages, and measures of central tendency were computed. The bivariable analysis was also carried out to inspect the association between the dependent and each independent variables. All independent variables that were statistically significant at the bivariable model < 0.2 *p*-values were included in the multivariable logistic regression model to prevent the possible effects of a confounder. Adjusted odds ratios with a 95% confidence interval using the enter method were performed and variables with p-value < 0.05 in the multivariable model were considered as statistically significant.

#### Spatial analysis

Geographical Information System (ArcGIS version 10.4) software was used to visualize maps and analyze spatial statistics. Global and local scale spatial autocorrelation analysis were applied to explore the presence of clustering in the area and to detect the geographical location of clusters of the early age sexual initiation. The Moran’s I index is the correlation coefficient, which measures the degree of association between a single variable with itself at different points in space as a function of the distance between points. The global Moran’s I statistic was used to measure the geographical clustering over the nation; whereas local Moran’s I statistic is used for constructing a localized measure of autocorrelation [12].

#### Spatial autocorrelation analysis

According to Tobler’s first law of geography, “everything is related to everything else, but near things are more related than distant things [13, 14].” The spatial autocorrelation (Global Moran’s I) statistic measures were used to evaluate whether the case patterns are dispersed, clustered or randomly distributed in the study area in which its values range from − 1 to 1 where Moran’s I values close to − 1 indicate perfect negative spatial autocorrelation (case dispersed), whereas Moran’s I close to 1 means positive spatial (case clustered) and Moran’s I zero implies perfect spatial randomness [15].

Anselin Local Moran’s I was used to investigating the existence of local level cluster locations of early sexual initiation and Moran’s I measure whether there were positively correlated (high-high and low-low) clusters or negatively correlated (high-low and low-high) clusters which are called outliers [16].

#### Hot spot analysis (Getis-Ord Gi* statistic)

The hot spot analysis tool calculates the Getis-Ord Gi* statistic that produces z-scores and *p*-values at a confidence level less than 0.05 which tell us where features with either high or low values cluster detected were statistically significant or not. Z-scores were used to assess the statistical difference of geographic clustering of early sexual initiation. A high (positive) z-score and small *p*-value of a feature indicate a significant hot spot whereas a low (negative) z-score with a small p-value indicates a significant cold spot; the higher or lower the z-scores, the more stronger the clustering, and a z-score near zero means no spatial clustering [17].

#### Spatial interpolation

Spatial interpolation technique was applied to predict values at unknown (non-sampled) locations using values at the measured (sampled) locations [18, 19]. Kriging spatial interpolation method was applied for predictions and produce smooth surfaces of the early age of sexual initiation.

#### Cluster detection and spatial scan statistical analysis

The spatial Scan statistical method is widely recommended since it performs very well in detecting local clusters [20]. It tests the presence of statistically significant spatial hotspots or clusters of early sexual initiation using Kuldorff’s SaTScan version 9.4 software. It uses a scanning window that moves across a study area; women who started sexual intercourse before the age of 18 were considered as cases and those the age group of 18 and above as controls to fit the Bernoulli model [12, 21].

Spatial cluster size < 25% of the population was used, as a higher boundary, which allowed both small and large clusters to be detected and ignored clusters that contained more than the maximum boundary. For each potential cluster, a likelihood ratio test statistic was used to determine if the number of observed early age initiation of sexual intercourse within the potential cluster was significantly higher than expected or not. The primary and secondary clusters were identified and assigned *p*-values and ranked based on their log likelihood ratio test, on the basis of 999 Monte Carlo replications [12, 21].

#### Ethical consideration

This study was based on an analysis of existing survey data with all identifier information that can be linked to particular individuals were removed. Written consent was obtained from the Measure DHS International Program, which authorized the datasets.

## Results

### Socio-demographic characteristics

From the total 12,033 women who participated in the study, a higher number of participants 37.6 and 24.8% were from Oromia and Amhara regions respectively. Four thousand nine hundred seventy-eight (41.4%) of respondents’ age ranges from 25 to 34 years and the mean age of the respondents was 30.9 years (SD ± 8.3). Of the total respondents, 5235 (43.5%) were Orthodox Christians followed by Muslim 3945 (32.8%) by religion. More than half 7095 (59%) of women didn’t have any formal education from which rural residence constitute the major share of 6576 (92.8%). Most of the respondents (84.9%) were married. Slightly more than half of the respondents 6219 (51.7%) had some jobs for pay (Table 1).

**Table 1:** Socio-demographic characteristics of respondents, EDHS 2016 (weighted sample, *n* = 12033)

Variables	Residence		Total
	Urban n (%)	Rural n (%)	
Current age in years			
15–24	496 (17.4)	2366 (82.6)	2865 (23.8)
25–34	1067 (21.4)	3911 (78.6)	4978 (41.4)
≥ 35	766 (18.3)	3424 (81.7)	4190 (34.8)
Mean ± SD (30.74 ± 8.39			
Highest educational level			
No education	519 (7.3)	6576 (92.7)	7095 (59.0)
Primary	772 (22.1)	2727 (77.9)	3499 (29.1)
Secondary	552 (63.7)	315 (36.3)	867 (7.2)
Higher	489 (85.3)	84 (14.7)	573 (4.7)
Region			
Tigray	198 (22.6)	677 (77.4)	875 (7.3)
Afar	28 (25.7)	81 (74.3)	109 (0.9)
Amhara	519 (17.4)	2459 (82.6)	2978 (24.8)
Oromia	575 (12.7)	3946 (87.3)	4521 (37.6)
Somali	61 (17)	297 (83.0)	358 (3.0)
Benshagul gumuz	19 (14.8)	109 (85.2)	128 (1.1)
SNNPR	282 (12.0)	2074 (88.0)	2356 (19.6)
Gambela	17 (45.9)	20 (54.1)	37 (0.33)
Hararai	16 (53.3)	14 (46.7)	30 (0.2)
Addis Ababa	572 (100)	0	572 (4.8)
Dire Dawa	45 (67.2)	22 (32.8)	672 (0.6)
Religion			
Orthodox Christian	1416 (27)	3819 (73)	5235 (43.5)
Muslim	462 (11.7)	3483 (88.3)	3945 (32.8)
Protestant	431 (16.7)	2149 (83.3)	2580 (21.4)
Others	24 (8.7)	251 (91.3)	275 (2.3)
Wealth Index			
Poorest	64 (2.8)	2190 (97.2)	2254 (18.7)
Poorer	22 (1.0)	2291 (99.0)	2313 (19.2)
Middle	35 (1.5)	2318 (98.5)	2353 (19.6)
Richer	87 (3.8)	2210 (96.2)	2297 (19.1)
Richest	2133 (75.4)	693 (24.6)	2816 (23.4)
Respondent Occupation			
Not working	765 (13.2)	5049 (86.8)	5814 (48.3)
Working	1567 (25.2)	4652 (74.8)	6219 (51.7)
Marital status			
Single	233 (58.1)	168 (41.9)	401 (3.3)
Married	1655 (16.2)	8562 (83.8)	10,217 (84.9)
Widowed	123 (28.7)	306 (71.3)	429 (3.6)
Divorced	1250 (33.1)	506 (66.9)	756 (6.3)
Separated	71 (30.7)	160 (69.3)	231 (1.9)

### Sexual intercourse characteristics of study participants

**Fig. 2:**
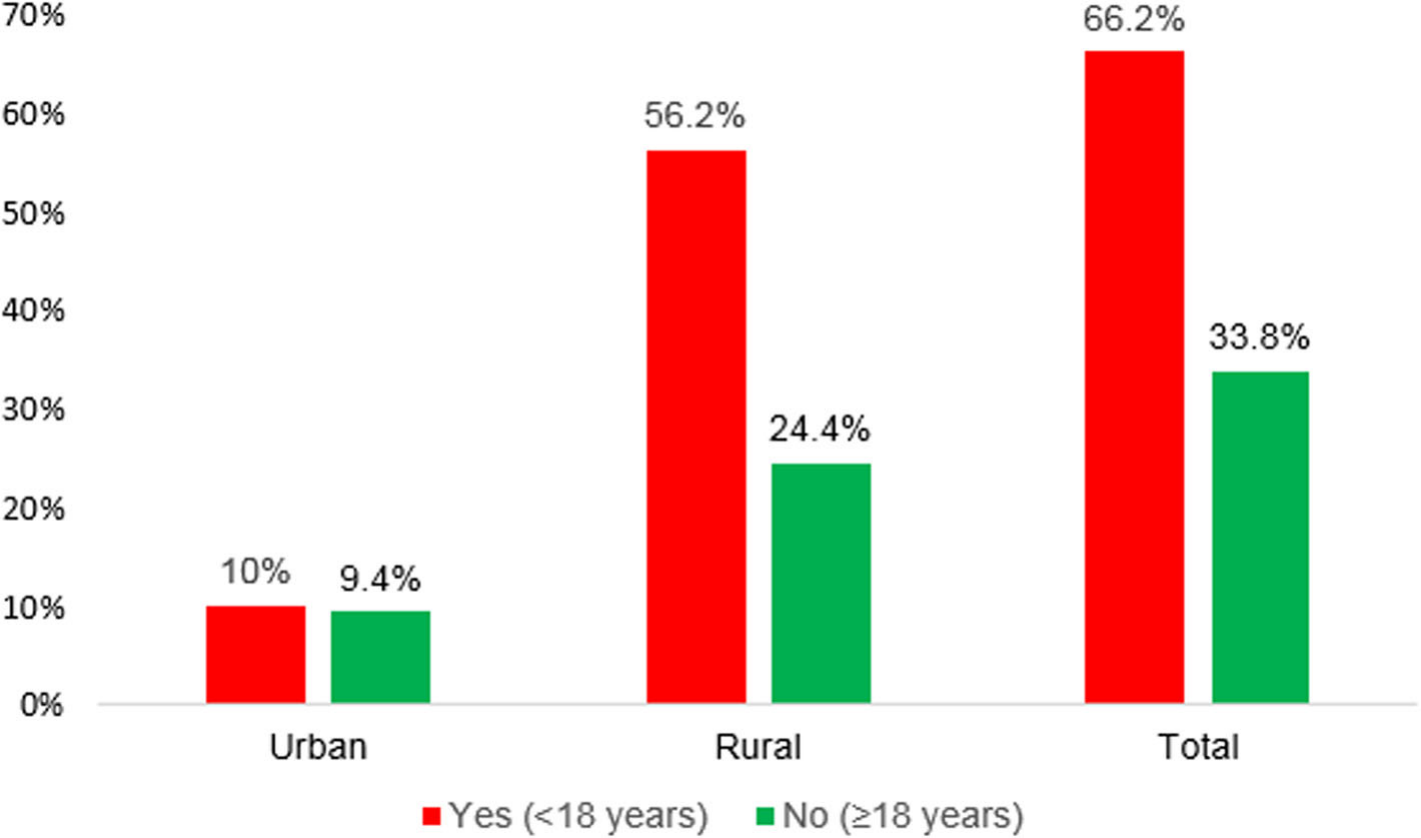
Prevalence of early age sexual initiation among women in Ethiopia, EDHS 2016

After weighing the sample, the proportion of early sexual initiation of the respondents was 66.2% (95% CI: 65.35, 67.05%) (Fig. 2). The median age of first sexual intercourse before the age of 18 was 16 years (±3.3) year. The age difference of respondents for their first sexual intercourse ranges from 8 years to 35 years. Among the respondents (*n* = 10,586) who gave birth, 4449 (42%) of them were before the age of 18 and rural area women took the major share of the first birth proportion 3833 (86.2%) and 2106 (94.4%) of rural areas women have had more than seven children. Regarding their current pregnancy status, 1235 (10.3%) of them checked and were sure about their pregnancy status (Table 2).

**Table 2:** Sexual characteristics of reproductive-age women respondents, EDHS 2016 (weighted sample, *n* = 12033)

Variable	Residence		Total n (%)
	Urban n (%)	Rural n (%)	
Age at first birth (*n* = 10,586)			
< 18 years	616 (13.8)	3833 (86.2)	4449 (42)
18–24 years	921 (17.2)	4447 (82.8)	5368 (50.7)
≥ 25 years	231 (30.1)	538 (69.9)	770 (7.3)
Number of children ever born			
No child	563 (38.9)	884 (61.1)	1447 (12)
1–3 children	1242 (25.2)	3679 (74.8)	4921 (41)
4–6 children	402 (11.7)	3033 (88.3)	3435 (28.5)
≥ 7 children	124 (5.6)	2106 (94.4)	2230 (18.5)
Currently pregnant			
No or unsure	2171 (19.9)	8727 (80.1)	10,898 (90.6)
Yes	161 (14.2)	974 (85.8)	1135 (9.4)
Ever terminated pregnancy			
No	2073 (119.2)	8725 (80.8)	10,799 (89.7)
Yes	259 (21)	976 (79)	1235 (10.3)

### Geographic variation and spatial clustering of early age sexual initiation

**Fig. 3:**
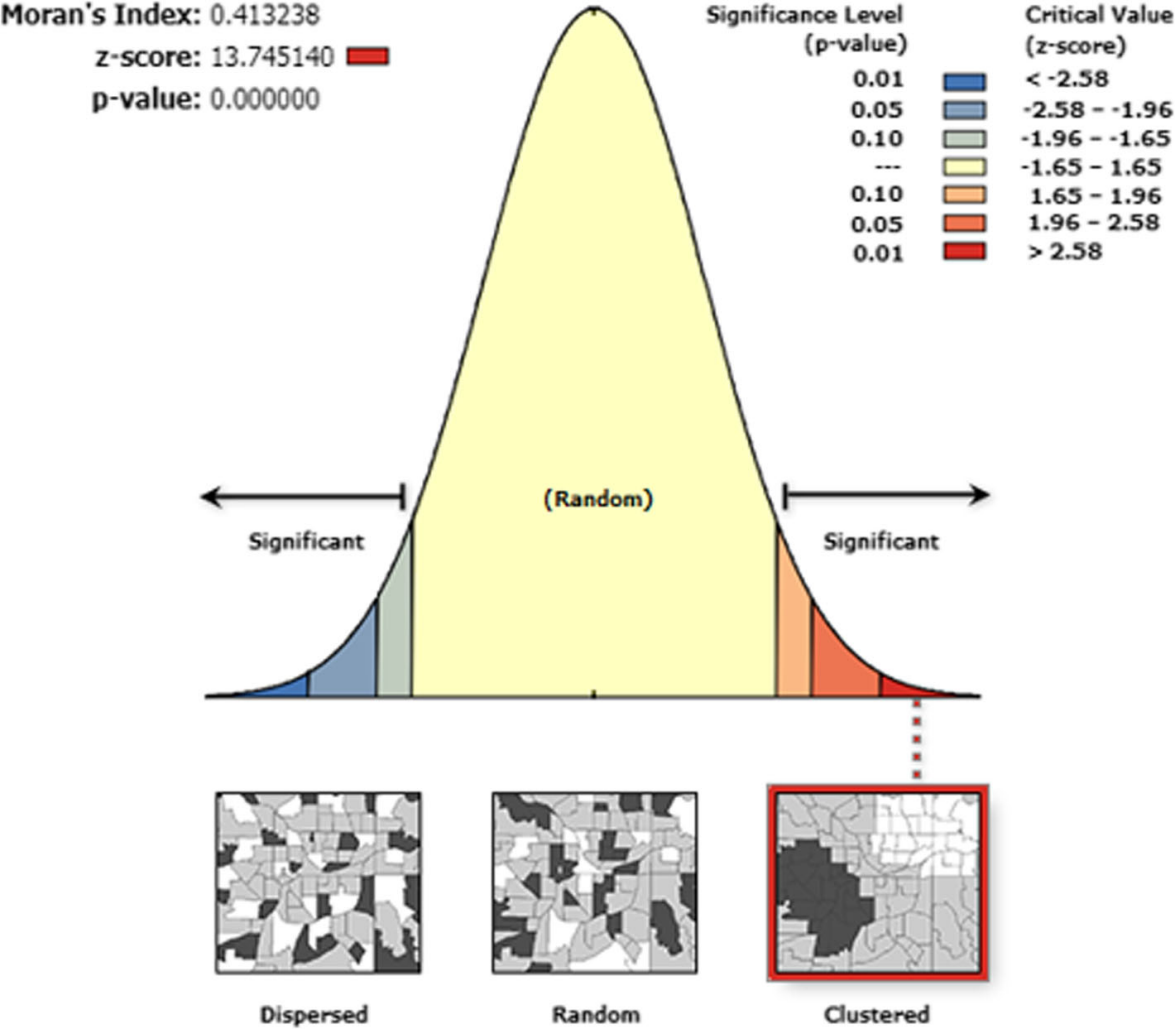
Spatial pattern of early sexual initiation among reproductive-age women in Ethiopia, EDHS 2016

In this study, geographical variation across regions for the early age of sexual initiation was observed. The spatial patterns of early sexual initiation of reproductive-age women were found to be non-random. The Global Moran’s I values (I = 0.41) indicated that there was significant clustering (99% confidence, *P* < 0.001) of early age sexual initiation in the country as a whole (Fig. 3).

**Fig. 4:**
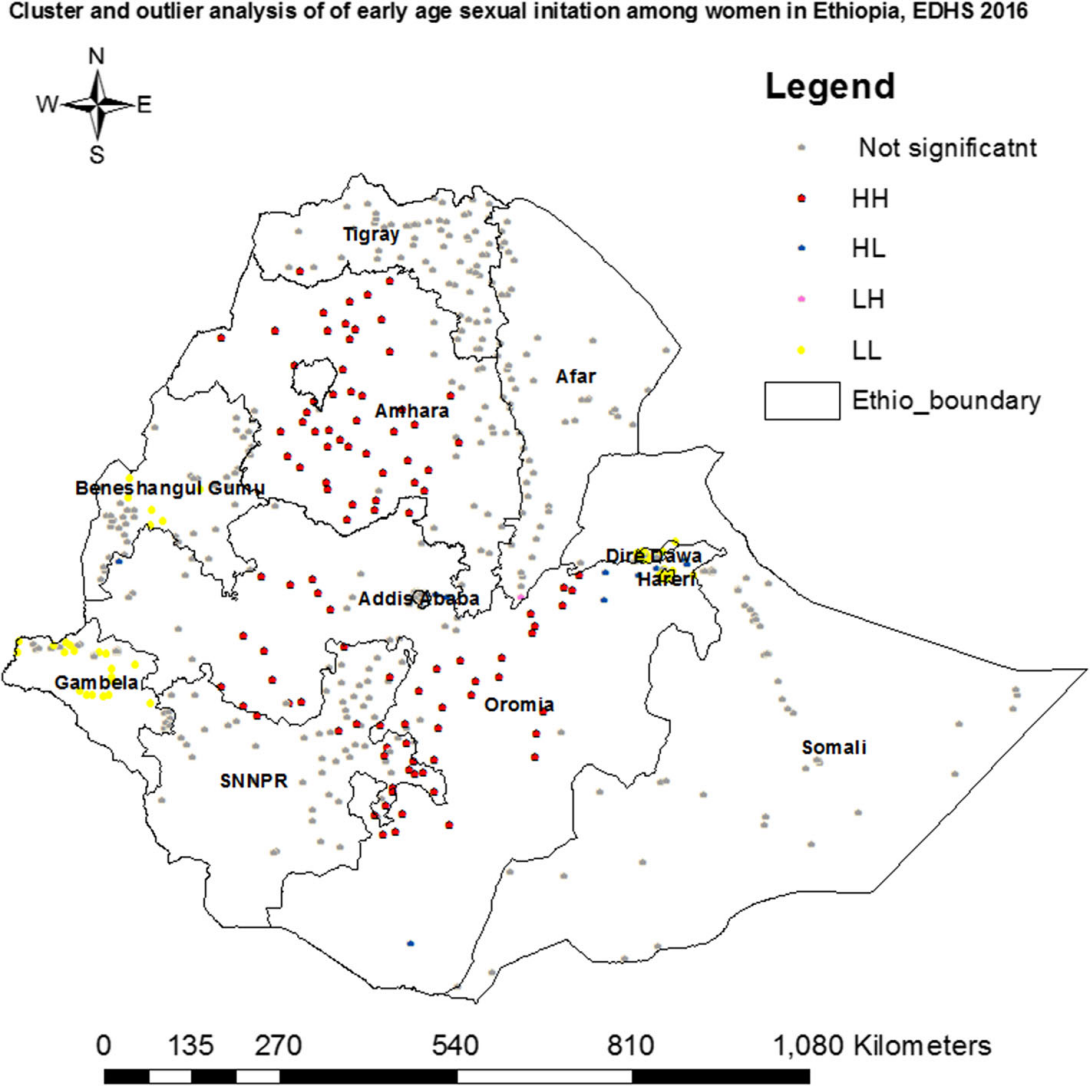
Cluster outlier identification of early age sexual initiation in Ethiopia, EDHS 2016

**Fig. 5:**
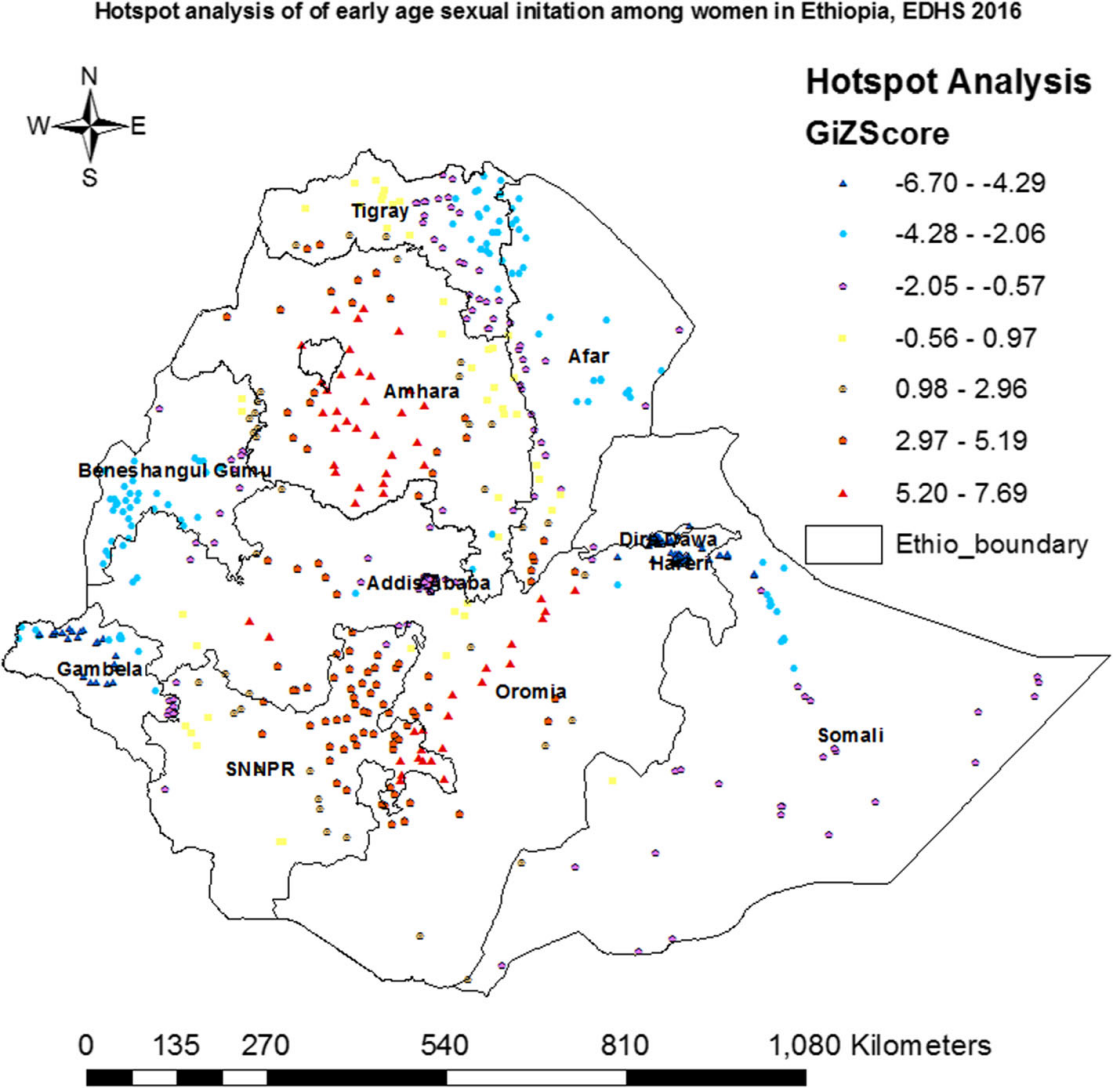
Hot spot identification of early age sexual intercourse in Ethiopia, EDHS 2016

Cluster and outlier analysis was used to detect hot spot areas and outliers of early sexual initiation. Hot spots were detected in most parts of Amhara and Oromia, Southwest Tigray, and Eastern part of Southern Nations, Nationalities, and Peoples’ Region (SNNPR) regions; whereas cold spots in Gambela, Benshangul-Gumuz, Dire Dawa, Harari and Addis Ababa regions. Similarly, outliers were also detected in Northwest Oromia, Addis Ababa, Harari and Dire Dawa (Fig. 4). In general, Getis-Ord Gi* was employed to check whether the detected clusters were statistically significant or not. Based on the result, high positive hot spots (Z-Score: 2.97–7.69, *p*-value < 0.001) were detected in most parts of Amhara and Oromia, Southern Afar, Northeastern SNNPR and Eastern Tigray regions; whereas high negative hotspots (Z-Score: − 2.06 to - 6.70, p-value < 0.001) were also detected Eastern Tigray, Central and Northern parts of Afar, Central and Northern parts of Gambela, Northern Somali, Central and Southern parts of Benshangul-Gumuz, Dire Dawa, Western Oromia, and Harari regions (Fig. 5).

### Spatial scan statistics result

In this study, data were representative at the national, regional, and rural-urban levels and can be generalized to all women early age sexual initiation in Ethiopia. The Geographic information system (GIS) and SaTScan statistical tests detected similar and statistically significant high-risk clusters/hotspots of early age sexual initiation. From the SaTScan output, we identified 14 spatial clusters, from which the first five of them were statistically significant at *P*-value < 0.05.

**Fig. 6:**
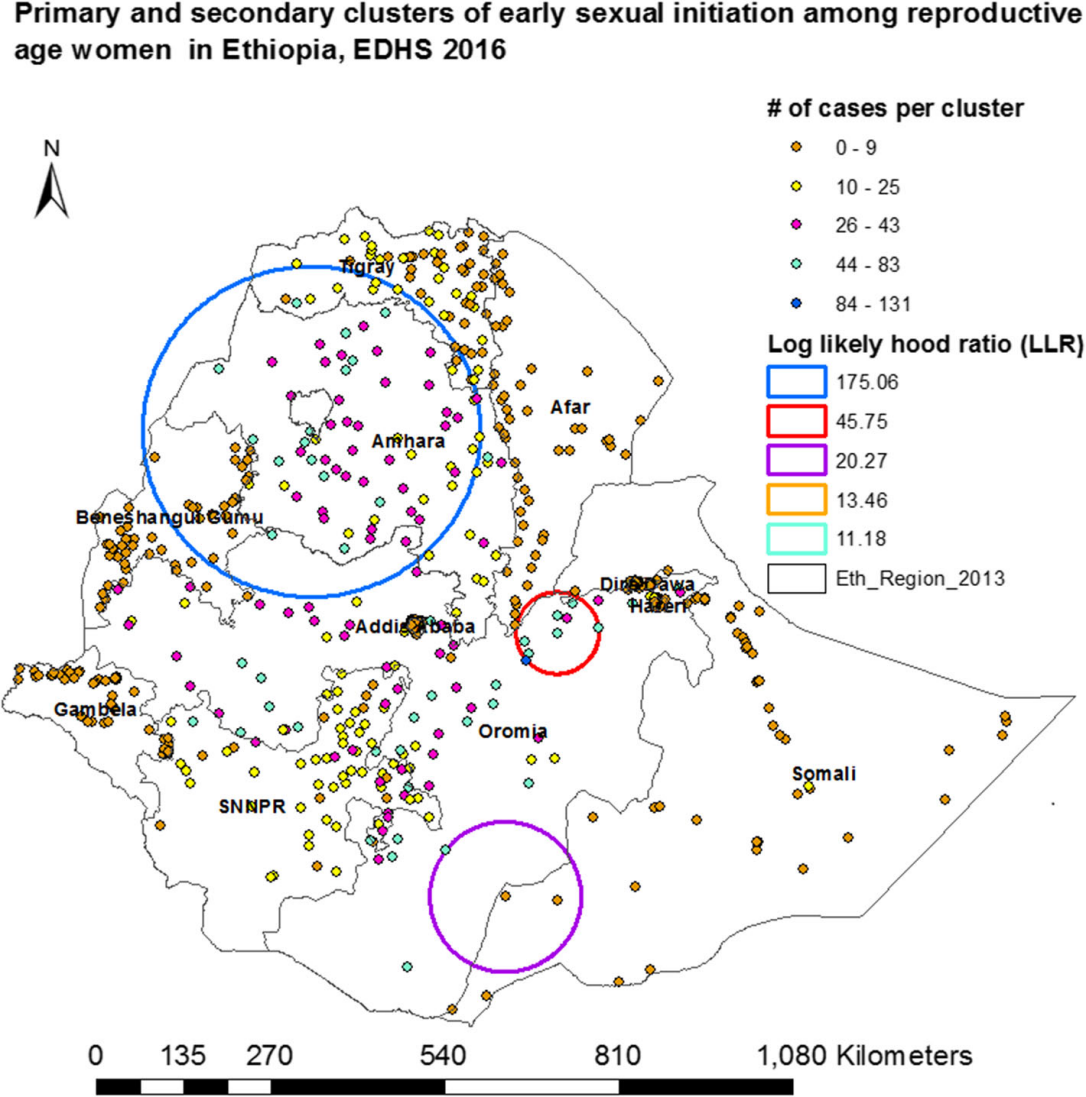
Primary and secondary clusters of early sexual initiation among reproductive-age women in Ethiopia, EDHS 2016

The primary cluster, the blue color ring spatial window was typically located at the Northern part of the country which encompasses most parts of Amhara, Northeast Benshangul-Gumuz, Southern Tigray and Northern parts of Oromia regions. This spatial window was centered at 11.699828 N, 37.313043 E with a 256.31 km radius. In addition, the remaining two spatial windows with red and pink colors were secondary clusters. The red color spatial window covers Northeastern Oromia, Western Dire Dawa, and Southern Afar whereas the pink color spatial window embraces Southern Oromia and Southwest Somali regions. These spatial windows were centered at 8.888553 N, 40.744565 E with 63.62 km radius and 5.203234 N, 40.019732E with 116.80 km radius respectively. However, the third and the fourth spatial windows of the detected secondary cluster were not plotted, because the clusters were detected only a single location identification (ID) (Table 3, Fig. 6).

**Table 3:** Significant clusters of early age sexual initiation among reproductive age women, EDHS 2016

Type of cluster	Total # of population	Total # of cases	RR	Cases (%)	LLR	Coordinates/Radius
Most likely cluster^a^	2866	2305	1.3	80.4	175.06	(11.699828 N, 37.313043E)/256.31 km
Secondary cluster^b^	620	516	1.27	83.2	45.75	(8.888553 N, 40.744565E)/63.62 km
Secondary cluster^b^	82	31	1.43	95.1	20.27	(5.203234 N, 40.019732 E)/116 km
Secondary cluster^b^	33	33	1.5	100	13.46	(7.146476 N, 37.651926 E)/ 0 km
Secondary cluster^b^	37	36	1.46	97.3	11.18	(7.747932 N, 36.027719 E)/ 0 km

^a^Primary cluster at *p* < 0.0001; ^b^Secondary cluster at *p* < 0.05; *LLR* log likelihood ratio; *RR* relative risks

### Spatial interpolation

**Fig. 7:**
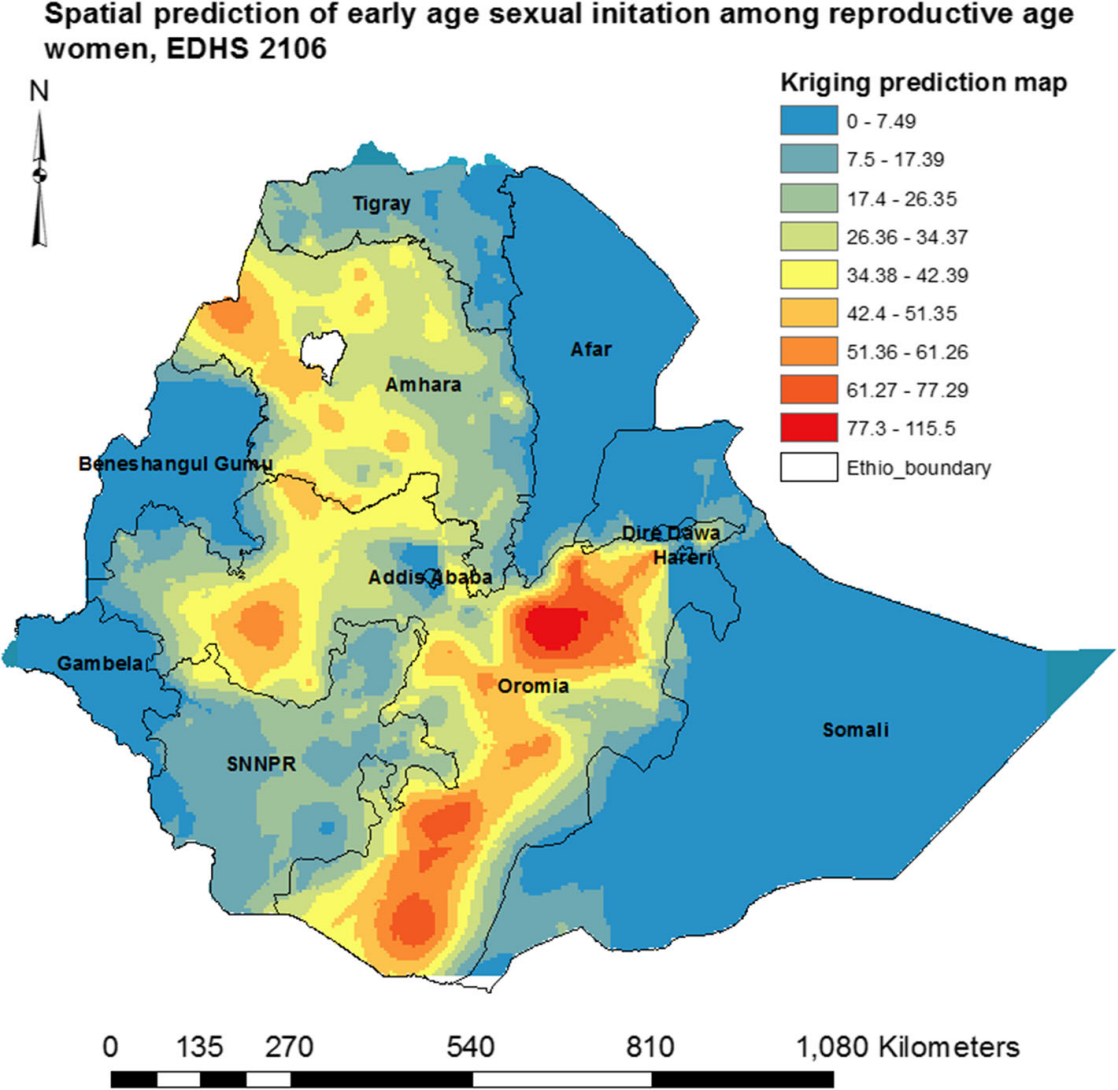
Spatial prediction of early age sexual initiation among reproductive-age women in Ethiopia, EDHS 2016

The kriging prediction map with dark red color told us that Eastern, Western and Southern parts of Oromia, Northwest and Southern parts of Amhara regions of the nation were predicted as risk areas for early age sexual initiation (Fig. 7).

### Factors associated with early sexual initiation among reproductive-age women

Early sexual initiation in Ethiopian women varied under the influence of various factors. After fitting the multivariable logistic regression, four variables were identified to be significantly associated with early age sexual initiation.

Being rural residence was found to be 1.31 times more likely to initiate sexual intercourse before the age of 18 years than urban women (AOR = 1.31, 95% CI: 1.11, 1.55). Married (AOR = 1.71, 95% CI: 1.36, 2.15), widowed (AOR = 2.63, 95% CI: 1.92, 3.61) and divorced (AOR = 2.32, 95% CI: 1.77, 3.06) women were 1.71, 2.63 and 2.32 times more likely to experience early sexual initiation as compared to single women respectively. Women with no education (AOR = 9.58, 95% CI: 7.60, 12.08), primary (AOR = 6.59, 95% CI: 5.25, 8.27) and secondary (AOR = 2.51, 95% CI: 1.96, 3.21) had 9.58, 6.59 and 2.51 times higher odds of having experienced early age sexual initiation as compared to higher educational level women respectively.

Poorer (AOR = 1.28, 95% CI: 1.12, 1.46) and richest (AOR = 1.46, 95% CI: 1.23, 1.74) were 1.28 and 1.46 times more likely to experience early sexual initiation when compared to poorest women respectively (Table 4).

**Table 4:** Factors associated with early age sexual initiation among reproductive-age women in Ethiopia, EDHS 2016 (weighted sample *n* = 12033)

Variables	Early sexual initiation		Crude OR (95% CI)	Adjusted OR (95% CI)
	No (%)	Yes (%)		
Residence				
Urban	1138 (28)	1194 (15)	Ref	Ref
Rural	2930 (72)	6771 (85)	2.20 (2.01, 2.42)	**1.31 (1.11, 1.55)**^a^
Wealth Index				
Poorest	702 (17.3)	1552 (19.4)	Ref	Ref
Poorer	627 (15.4)	1686 (21.2)	1.22 (1.07, 1.38)	**1.28 (1.92, 1.46)**^a^
Middle	716 (17.6)	1638 (20.6)	1.04 (0.91, 1.17)	1.12 (0.98, 1.27)
Richer	760 (18.7)	1537 (19.3)	0.92 (0.81, 1.04)	1.10 (0.97, 1.25)
Richest	1263 (31.0)	1552 (19.5)	0.56 (0.49, 0.62)	**1.46 (1.23, 1.74)**^a^
Marital Status				
Single	251 (6.2)	149 (1.9)	Ref	Ref
Married	3401 (83.6)	6816 (85.6)	3.37 (2.74, 4.15)	**1.71 (1.36, 2.15)**^a^
Widowed	109 (2.7)	321 (4.0)	4.96 (3.69, 6.68)	**2.63 (1.92, 3.61)**^a^
Divorced	209 (5.1)	547 (6.9)	4.41 (3.41, 5.70)	**2.32 (1.77, 3.06)**^a^
Separated	98 (2.4)	132 (1.7)	2.25 (1.62, 3.13)	1.31 (0.92, 1.86)
Education				
No education	1889 (46.4)	5206 (65.4)	10.50 (8.52, 12.94)	**9.58 (7.60, 12.08)**^a^
Primary	1214 (29.8)	2286 (28.7)	7.2 (5.79, 8.88)	**6.59 (5.25, 8.27)**^a^
Secondary	512 (12.6)	354 (4.4)	2.64 (2.07, 3.36)	**2.51 (1.96, 3.21)**^a^
Higher	453 (11.1)	119 (1.5)	Ref	Ref
Currently pregnant				
No or unsure	3634 (89.3)	7263 (91.2)	Ref	Ref
Yes	434 (10.7)	702 (8.8)	1.2 (1.1, 1.4)	1.0 (0.8, 1.1)
Ever had terminated				
No	3700 (91.0)	7098 (89.1)	Ref	Ref
Yes	368 (9.0)	867 (10.9)	1.2 (1.1, 1.4)	1.1 (0.9, 1.2)

Hosmer-Lemeshow Goodnes of Fit Test = 0.89; Ref = reference; ^a^ = Significant

## Discussion

In this study, the prevalence and spatial distribution of early age of sexual intercourse was assessed. The number of early sexual initiation cases per cluster was found to vary geographically) Five statistical significant spatial clusters (*P*-value < 0.01) were also detected across the regions of the country Findings from this study showed that, the prevalence of early sexual initiation among reproductive-age women was 66.2% (95% CI: 65.4, 67.1%). The result of this study was lower than a study conducted in South Africa 70.8% [22]. The difference, higher in South Africa may be explained by the study participant’s composition (African, White, and Asian). In addition, there was also higher proportion of urban residence (76.2%) respondents who participated in the study compared to the current study 19.4% which gave chance of getting access to education which might help them to increase their awareness of the negative consequences of early age sexual intercourse.

However, the current study proportion was higher than different pocket study findings conducted in Ethiopia (29% [11], 59.5% [23], 56.9% [24], 53.7% [10]), Nigeria 28.7% [25], China 10.7% [7] and Tanzania 57.8% [26]. The possible explanation for this might be due to the advancement of technology that enhances watching pornographic videos and substance use which may increase the probability of taking part in early sexual intercourse activities.

Our study recognized that place of residence, household wealth index, educational attainment, and marital status as statistical significant contributing factors for early sexual initiation in the multivariable logistic regression analysis. From the 1136 (9.4%) currently pregnant and 1235 (10.3%) ever had a history of terminating pregnancy respondents; 702(61.8%) and 867(70.2%) of them started early age sexual intercourse respectively although they were not statistically significant in the multivariable logistic regression analysis (Table 4).

Rural residence respondents were 1.31 times more likely than urban area women for early sexual initiation [AOR = 1.31, 95% CI: (1.11, 1.55)]. This finding was in line with pocket studies conducted in Ethiopia [8, 9]. The possible explanation for this could be low awareness of the community on reproductive health issues and the bad consequence of early marriage for rural adolescents.

Another significant association was also maintained between marital status and early sexual initiation. Being widowed [AOR = 2.63, 95% CI: (1.92, 3.61)], divorced [AOR = 2.32, 95% CI: (1.77, 3.06)] and married [AOR = 1.71, 95% CI: (1.36, 2.15)] were 2.63, 2.32 and 1.71 times more likely to experience early sexual initiation when compared to never married respondents respectively. This finding is comparable to pocket studies done in Ethiopia [9, 10, 27]. The possible justification for this might be engaged in early marriage (before the age of 18 years) which is the potential scenario for women to be engaged in early age sexual intercourse activity. As indicated on the EDHS 2011 report, the median age of first marriage in the country was 17.1 years [11].

A strong statistical association was also held between education and early sexual initiation. Women with no education [AOR = 9.58, 95% CI: (7.60–12.8)], primary [AOR = 6.59, 95% CI: (5.25–8.27)] and secondary [AOR = 2.51, 95% CI: (1.91–3.21)] had 9.58, 6.59 and 2.51 times higher odds of having experienced early age sexual initiation as compared to women having higher educational level respectively. This finding is consistent with a study done in Ethiopia [9, 28, 29], Malawi [30], Kenya [29], South Africa [31] and Nigeria [32]. The possible explanation for this could be when women had better educational status, they will understand potential risks related with early age sexual intercourse and might protect themselves from being engaged.

Another statistical significant association was also identified between wealth index and early age sexual initiation. Poorer [AOR = 1.28, 95% CI: (1.12–1.46)] and richest [AOR = 1.46, 95% CI: (1.23, 1.74)] were 1.28 and 1.46 times more likely to experience early sexual initiation as compared to the poorest women respectively. This finding is consistent with studies done in Kenya [29]. The probable reason for this could be an economic problem for poorer ones; economically poor respondents might be cheated by gift (either in cash or in-kind) which initiates them to be volunteer to take part. On the other hand richest respondents as they were capable of financial issues, they might frequently attend different pornographic films using different technological products which pushes them to be involved in early sexual intercourse.

The spatial pattern was non-random and hot spots areas were found in most parts of Amhara and Oromia, Southwest Tigray, and Eastern part of Southern Nations, Nationalities, and Peoples’ Region (SNNPR) regions. On the other hand, hot spot areas were found in the regions where majority of the populations reside. The study shows that significant geographical variations in early age sexual initiation exists within Ethiopia. This study finding is consistent with studies conducted in Nigeria [33].

However, there were some limitations in this study. Sexual practice has private, intimate, and sensitive nature in the society, there might be an underreporting of some behaviors. Similarly, self-reporting of sexual behaviors could have introduced recall or social desirability bias. The cross-sectional nature of the data also prevents causality from being inferred between age of sexual initiation and significant predictors. In addition, respondents’ data who didn’t have files (longitude and latitude) were excluded from the spatial analysis which could affect the overall result and the generalization we made as well.

Despite, SaTScan analysis can identify spatial clusters, but can’t explain why the variations in the risks of the events of interest exist.

## Conclusion

In general, the prevalence of early age sexual initiation among reproductive-age women was high. Residence, educational attainment, wealth index, and marital status were identified as statistically significant factors. A total of five significant clusters (most likely and secondary clusters) (LLR = 175.06, 45.75, 20.27, 13.46 and 11.18, at *P*-value < 0.01) were detected in Amhara, Oromia, Somali, Tigray and Benshangul-Gumuz regions of the country. Therefore, identifying the hot spot areas for early sexual intercourse can help to design tailored innervations which can help to reduce maternal morbidity and mortality contributed by early sexual intercourse.

## Data Availability

The data in which the authors used to produce this manuscript are available and the authors are prepared to share our data on request recognizing the benefits of such transparency. Otherwise, the data-set can be accessed through www.dhsprogram.com after subscription and being an authorized user.

## Abbreviations

CSA: Central Statics Agency
EDHS: Ethiopia Demographic and Health Survey
GIS: Geographic Information System
GPS: Global Positioning System
LLR: Log Likelihood Ratio
OR: Odds Ratio
RR: Relative Risk
SNNPR: Southern Nations, Nationalities, and Peoples’ Region
